# Diving head-first into brain intravital microscopy

**DOI:** 10.3389/fimmu.2024.1372996

**Published:** 2024-05-16

**Authors:** Althea R. Suthya, Connie H. Y. Wong, Joshua H. Bourne

**Affiliations:** Centre for Inflammatory Diseases, Department of Medicine, School of Clinical Sciences at Monash Health, Monash Medical Centre, Monash University, Clayton, VIC, Australia

**Keywords:** intravital microscopy, imaging, brain, stroke, neuroinflammation

## Abstract

Tissue microenvironments during physiology and pathology are highly complex, meaning dynamic cellular activities and their interactions cannot be accurately modelled *ex vivo* or *in vitro*. In particular, tissue-specific resident cells which may function and behave differently after isolation and the heterogenous vascular beds in various organs highlight the importance of observing such processes in real-time *in vivo*. This challenge gave rise to intravital microscopy (IVM), which was discovered over two centuries ago. From the very early techniques of low-optical resolution brightfield microscopy, limited to transparent tissues, IVM techniques have significantly evolved in recent years. Combined with improved animal surgical preparations, modern IVM technologies have achieved significantly higher speed of image acquisition and enhanced image resolution which allow for the visualisation of biological activities within a wider variety of tissue beds. These advancements have dramatically expanded our understanding in cell migration and function, especially in organs which are not easily accessible, such as the brain. In this review, we will discuss the application of rodent IVM in neurobiology in health and disease. In particular, we will outline the capability and limitations of emerging technologies, including photoacoustic, two- and three-photon imaging for brain IVM. In addition, we will discuss the use of these technologies in the context of neuroinflammation.

## Introduction

Some say every good story starts with a humble beginning. This was indeed the case for the discovery of intravital microscopy (IVM). Augustus Volney Waller (1816–1870) took advantage of the elasticity and transparency of the frog tongue which enabled him to observe leukocyte emigration by brightfield microscopy within a living organism (1). Twenty years later, Julius Friedrich Cohnheim (1839–1884) utilised IVM to identify that the cells lining the vessels facilitated recruitment of leukocytes, and he described how leukocytes can transmigrate from the vessel to enter tissues (2). These early breakthroughs relied on white-light tissue illumination coupled with the simplest method of optical microscopy. Over the past centenaries, the techniques of IVM have been refined on tissues from various animal species to facilitate discoveries relevant to human health and disease.

Transillumination IVM of cremaster muscle was instrumental in describing the molecular basis which underlies leukocyte recruitment and adhesion during inflammation (3). Additionally, the unique anatomical characteristics of the mesenteric vasculature enabled the *in vivo* study of thrombosis and hemostasis through live imaging in mammals (4). Whilst few organs share the transparent nature of the cremaster muscle, major arteries and organs such as the liver and kidney are easily accessed and imaged by intravital microscopy with trained surgical technique (5–8). Unlike the aforementioned tissues and organs, the mouse brain is encapsulated by a 200 micron-thick skull, which is critical to neuronal protection and function. In the context of neurobiology, the skull was a major obstacle for light penetration to visualise cerebral vasculature. This was first overcome with the implantation of an air-tight cranial window directly above the pia matter to observe vasculature by light microscopy in live mammals, which was first described by Donders in 1850, later optimised by Forbes (9), and recently reviewed by De Niz et al. (10). Traditionally, most brain IVM studies require the rodent to receive a craniotomy or at least thinning part of the skull to improve image resolution. Such surgical manipulation would be performed at the time of imaging thus unlikely to significantly impact the acute neurobiological processes of interest. However, implantation of a cranial window for chronic studies would inevitably alter otherwise naïve physiology. This is in combination with anaesthetic necessary for surgery and imaging, which in itself mounts an acute immune response (11). This is particularly concerning with recent discovery that the leukocytes from the skull (calvarium) bone marrow participate in neurological health and disease (12, 13). Fortunately, advancement in microscopy technologies and new strains of genetically modified reporter mice that express endogenous fluorescent proteins have allowed for imaging cell-cell interactions in the brain at unprecedented resolution, few without the need for cranial manipulation or window implantation. It must be indicated that whilst microscopy is semi-quantitative, it should be complemented with other techniques to confirm a biological event. For example, cell number can be assessed by multicolour flow cytometry analysis (14), and blood brain barrier (BBB) integrity can be assessed by leakage of large compounds such as Evans blue (15). In this review, we will outline some of these microscopy technologies, and their respective advantages and limitations. Additionally, we will discuss the use of brain IVM and associated tools in studying the behaviours and migrations of different cerebral cell types in the context of neuroinflammation.

## IVM: the hardware

**Figure 1:**
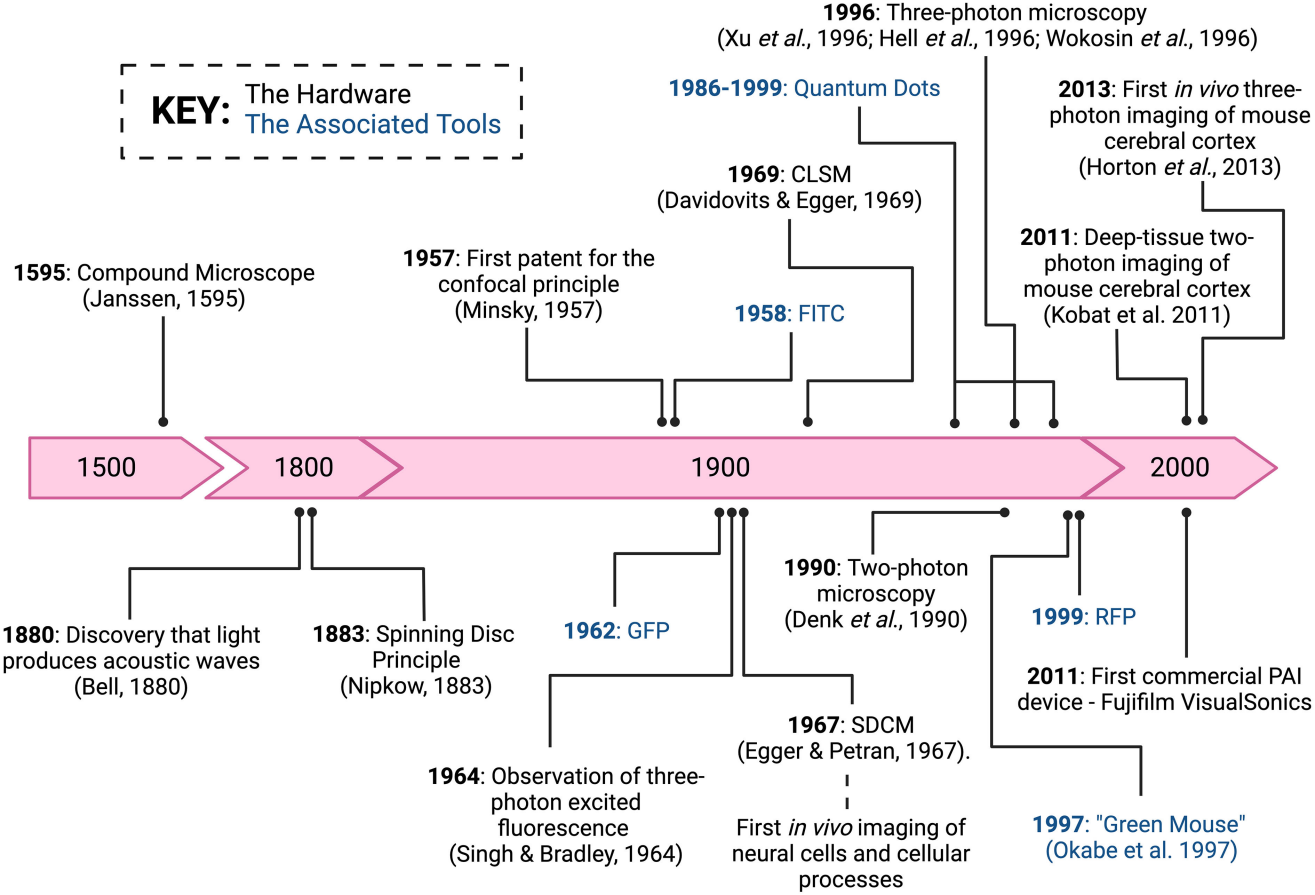
Intravital microscopy significant discoveries and developments. A timeline of major discovery milestones of the hardware and associated tools of brain intravital imaging. Image created with BioRender.com.

Single-photon microscopy employs epifluorescence and forms the basis of fluorescent widefield and confocal microscopes. It involves the light illumination and excitation to travel through an objective lens, where the image is a product of fluorescence emitted from the tissue recognised by detectors at specific wavelengths. Compared to brightfield microscopy, this technology has significantly improved the imaging depth and resolution. Here, we will specifically focus on microscopy techniques more commonly used for brain IVM, those that have garnered potential within the field, and the respective advantages and disadvantages (**Table 1**) of each. **Figure 1** depicts a timeline of significant discoveries and development of brain IVM in rodents.

**Table 1:** Imaging techniques. The advantages, disadvantages, and generalised specification of common microscope hardware.

		Resolution	Depth Penetration	Image Acquisition Speed	Phototoxicity	Cost	Operation/Technical Complexity	Require Labelling Probes?
**Transillumination**	**Brightfield**	Low	Low	Fast	High	$	Low	Optional
**Single-Photon**	**Widefield**	Low	Low	Fast	High	$	Low	Yes
	**Confocal**	Decent - High	Average	Slow (CLSM) Fast (SDCM)	Low	$$	Average	Yes
**Multiphoton**	**2P**	High	High	Slow	Low	$$$	Average - High	Yes
	**3P**	High	High	Slow	Low	$$$$	Average - High	Yes
**Photoacoustic Imaging**	**OR-PAM**	High	Low	Average	Low	$$$	Average	No
	**AR-PAM**	Average	Very High	Fast	Average	$$$	Average	No

CLSM, Confocal Laser Scanning Microscopy; SDCM, Spinning Disk Confocal Microscopy; 2P, 2-Photon; 3P, 3-Photon; SHG, Second-Harmonic Generation; THG, Third-Harmonic Generation; OR, Optical Resonance; AR, Acoustic Resonance; PAM, Photoacoustic Microscopy.

### Confocal microscopy

**Figure 2:**
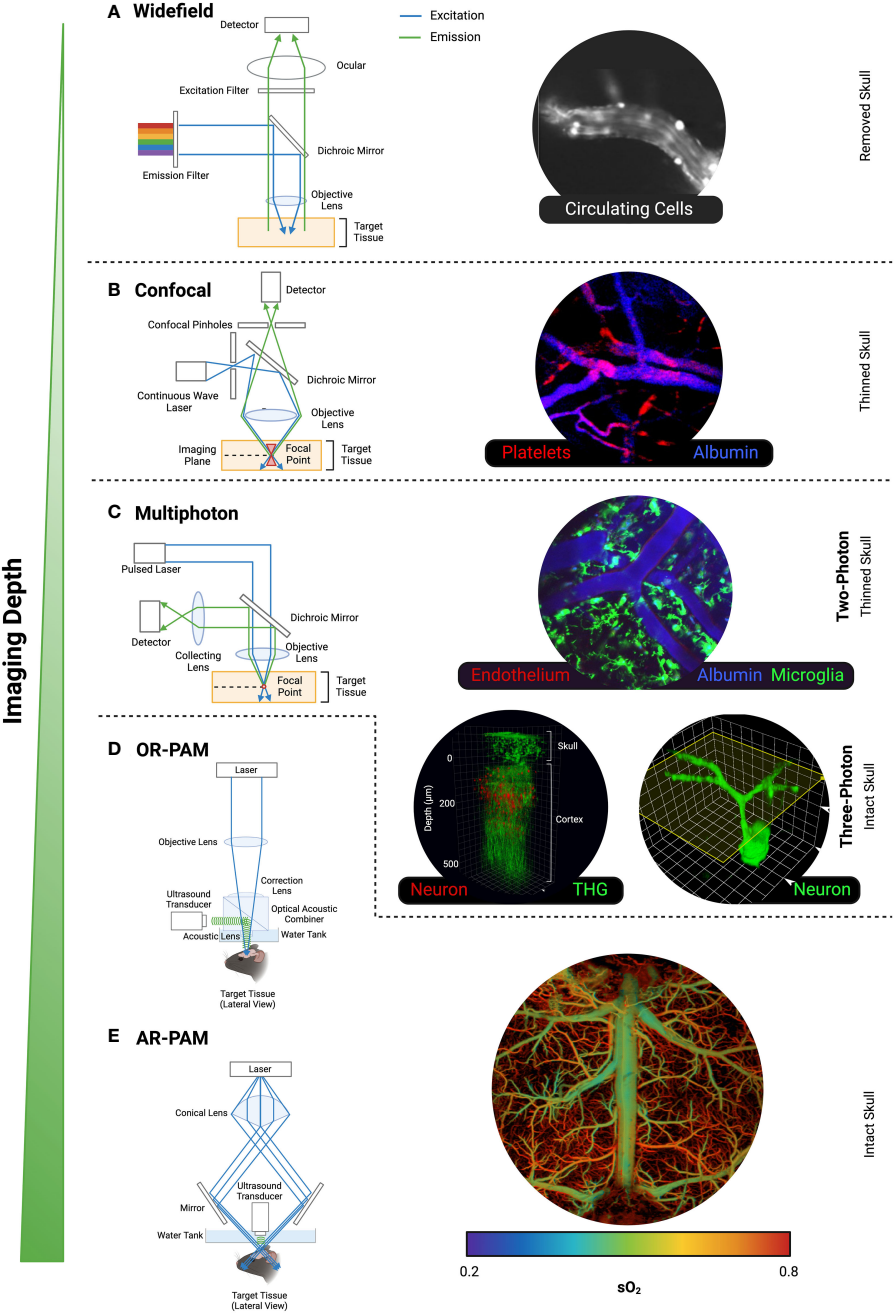
Common intravital microscopes. The light excitation and emission pathways, and intravital images of mouse brain taken from **(A)** Widefield Microscopy, **(B)** Confocal Microscopy, **(C)** Multiphoton Microscopy, **(D)** OR-PAM, and **(E)** AR-PAM. The cell type or label used are in the corresponding colour font for each image. Representative images were our own work (confocal and 2PM) or adapted from published work with permission [Wong et al. for Widefield (16); Wang et al. for 3PM (17); Zhu et al. for AR-PAM (18)]. Created with BioRender.com.

To avoid capturing out-of-focus emission light, as seen with bright- and widefield microscopy (**Figure 2A**), the uniqueness of a confocal microscope is in its pinhole design (**Figure 2B**). Since its first patent in 1957 (19), variations of confocal microscopy have been developed (20–22). Perhaps the most pivotal event in not only confocal microscopy, but fluorescent microscopy history was the development of confocal laser-scanning microscopes (CLSM). CLSM utilises a single pinhole, and although this is a powerful tool in providing high-resolution images, its slow point-by-point scanning method generally limited the use of this imaging technique to fixed-tissue samples or slow-moving cells *in vivo*. In light of the relatively long duration of image acquisition, CLSM is less favourable in capturing biological events in real-time. As such, spinning-disk confocal microscopy (SDCM) is the more popular choice for intravital imaging. As the name suggests, SDCM utilises a fast-spinning opaque disk which consists of thousands of holes to increase area of specimen excitation simultaneously, resulting in low power but rapid imaging. Indeed, SDCM was once used to image unstained neuronal cells in salamander *in vivo* and ganglion cells from frogs but has since been developed to facilitate observation of cell fragments (platelets) within a micron scale *in situ, in vivo* (23). Overall, it is evident that confocal microscopy has been key in live imaging of cerebral events that enable our better understanding of neurobiology due to its speed, resolution and cost effectiveness. Despite this, it is important to note that the biological activities examined via this type of imaging technology are limited to the pial microvasculature on the surface of the brain as penetration depth is limited (24). Therefore, other microscopy techniques present greater advantages in imaging deeper brain tissue that perhaps would reveal cell-cell interactions that are more biologically relevant.

### Multiphoton microscopy

Multiphoton microscopy (MPM) traditionally refers to two-photon microscopy (2PM) and the associated instruments and techniques. However, with the emergence of three-photon microscopy (3PM), MPM has grown to become an all-encompassing umbrella term for more than one photon for excitation. Two-photon microscopy [2PM (25)] occurs from the simultaneous absorption of two photons, twice the wavelength of what is normally required for a single-photon fluorescent excitation. It has the same fundamental components as confocal microscopy except two key differences: a) use of a high powered, tuneable pulsed laser and b) absence of the “pinholes” (**Figure 2C**). Compared to confocal microscopy, the higher wavelength (typically in the infrared spectrum) and lower energy light in MPM allow for deeper tissue penetration, as the corresponding wavelengths are less scattered by tissue (26). Routinely, 2PM has capabilities of up to ~500 μm tissue penetration (27) and reduced photo bleaching, hence is therefore the preferred microscopy technology for brain IVM.

Additionally, rather unique to MPM is second harmonic generation (SHG), which is created from the interaction of light with non-centrosymmetric structures. SHG has since been found to have quite specialised applications in imaging microtubules (28), collagenous tissue structures (29) and myosin (30) without the use of fluorescent labels and stains, nor transgenic reporter animal models. As the role of collagen in the central nervous system (CNS) has only emerged recently (31, 32), there are currently limited studies that have used *in vivo* SHG to image cranial collagenous structures. However, using SHG in brain IVM has promising potential in new discoveries for neurodegenerative diseases such as Alzheimer’s disease (AD) or Parkinson’s disease (PD) as fibrinogen deposits contributes to neurodegeneration (33). Third harmonic generation (THG) is generated from the interaction of intense light with certain tissue matter refers to a light wave that is triple the frequency, but one-third of the original wavelength (34). Similar to SHG, THG generated by water-lipid and protein complexes in tissues of mammals has promising uses in performing label-free optical biopsies in clinical settings (35). Though THG is limited to tissue regions where there are significant changes in refractive index (speed at which light travels through a medium), it has been demonstrated that lipid bodies are a strong contrast source in THG (36). This is particularly advantageous as brain tissues, including axons and dendrites, have a high lipid concentration (37). Moreover, optical microscopy with higher-harmonics has also demonstrated its potential in long-term *in vivo* research by combining SHG and THG to observe the vertebrate embryonic nervous system development (37).

Multiphoton microscopy has undoubtedly revolutionised research by providing *in situ* visualisation of cell behaviour and interactions in real-time – a true testament of “seeing is believing”. Unlike the confocal microscope, the 2PM has no ‘out of focus’ fluorescence, therefore there is no signal-to-background ratio (SBR), providing a greater imaging depth (26, 38). SBR calculates the proportion of excitation energy that is absorbed against those that get scattered, and a loss of this ratio in a 2P microscope allows for imaging depth of up to 500 μm for brain IVM. As such, one strategy has been to increase excitation wavelengths to compensate, or counter any ‘weakening’ of absorption that may occur in tissues (26, 39). Albeit resulting in successful imaging depths of up to 1.6 mm in the mouse cortex (26), this imaging depth was achieved after complete removal of the skull which lessens the biological relevance. In order to achieve greater depth penetration in the brain and other organs, the 3PM was developed. The first demonstration of 3PM shortly followed 2PM (40–42), but only recently garnered more use within cerebral *in vivo* imaging. A potential upper hand of 3PM against 2PM is the reduced out-of-focus background, and thus increasing and improving SBR.

Optical *in vivo* imaging through the intact mouse skull is always going to be a challenge due to skull-induced light aberrations and scattering. In the past decade, the development of 3PM allowed for longer excitation wavelengths, enhanced background suppression by higher-order nonlinear excitation, and greater imaging depth with limited light scattering. Using this technique, brain IVM studies have revealed structure and function in the mouse hippocampus through cranial windows in intact brains (43, 44). Furthermore, recent advances in brain IVM with 3PM combined with a synthesised dye of unusually large three-photon excitation (3PE) cross-section at 1,550 nm enabled cerebral vasculature imaging through intact mouse skull that reached a depth of 300 μm below the skull (45). Not to be outdone, the latest advances in brain IVM with 3PM imaging of brain vasculature through the adult mouse skull has achieved above 500 μm in depth, reaching cortical layers 2/3 and 4 in awake mice (17). The neocortex is comprised of 6 layers of increasing depth, each of which contain neuronal pathways of differing function. Access to each layer is critical in brain IVM, however the definition of boundaries between layer 2 and 3 are blurred in rodents, and are so often labelled layer 2/3 (46). Indeed, progress in optical brain IVM does not only focus on imaging depth, but also on the ability to visualise neuronal events in freely behaving mice. Mice actively sense their environment using vision, thus a head-mounted MPM that could shift imaging depth at will between visual cortical layers would be ideal to study their full sensory repertoire. To this end, a head-mounted 3PM was developed to elegantly reveal neuronal activity in layer 4 and 6 of the mouse visual cortex in differentially modulated light/dark conditions during free exploration (47). Undoubtedly, the innovative designs and advancements in optical brain *in vivo* imaging has provided impactful insights into neurobiology. Congruently, there have been developments to image beyond the optical space, thus propelling the field into harnessing the power of photoacoustic energy.

### Photoacoustic imaging

The limited depth and resolution drawbacks of pure optical imaging have been improved through its coupling with acoustic detection, namely, photoacoustic imaging (PAI). Where ultrasound imaging (USI) depends on the rate of reflected ultrasound waves to determine a tissue’s consistency (48), the underlying principle of PAI is that when light from a pulsed laser gets absorbed, the heating of the target tissue releases pressure in the form of detectable acoustic waves. Incidentally, acoustic waves scatter far less than light, which facilitates imaging depths of centimetres (49), far exceeding multiphoton capabilities. Depending on the method of image formation, PAI can be divided into scanning-based photoacoustic microscopy (PAM) and reconstruction-based photoacoustic computed tomography (PACT). The raster (sawtooth-like wave pattern) scanning of the PAM generates an image, and the resolution is dependent on the focusing method of which the photoacoustic signal is emitted (i.e., acoustically or optically). As such, PAM is further distinguished as optical resolution PAM (**Figure 2D**; OR-PAM) and acoustic resolution PAM (AR-PAM; **Figure 2E**). The tightly focussed optical signal in OR-PAM achieves high resolution but subsequently has limited depth and field of view capabilities (50, 51). In contrast, the broader optical signal of AR-PAM allows imaging of deeper structures at a lower resolution (50). More recently, efforts have been made to advance the raster scanning of PAM into higher scanning speed alternatives. There are different hybrid approaches that have been adopted to minimise the compromise of detection sensitivity for higher scanning methods including piezo scanners (52, 53), galvo scanners (54, 55), and water-immersible microelectromechanical systems (MEMS) scanners (56, 57). Overall, PAI presents a more novel technique for *in vivo* brain imaging. Combining optical imaging with acoustic detection, PAI has gained attraction within brain research as a non-invasive and radiation-free method to investigate pathophysiology.

IVM technology has grown exponentially in recent times, however the future beyond 3PM resolution and PAM imaging depth is unclear. Whilst a 4-photon microscope exists, and has been implemented to image astrocytes by brain IVM (58), this microscope is not readily available to the community. It is expected that a combination of existing techniques will dominate the field for the time being.

## IVM: the associated tools

The efforts to advance the microscopy techniques for high-resolution *in vivo* imaging would be to no avail without fluorophore-tagged antibodies, fluorescent probes, and reporter animal models. Several IVM-associated tools have since developed to interrogate the identity of individual cell types recruited to the brain during neuropathology. As momentum built for the development and use of microscopes to reveal underlying pathophysiology of various biological mysteries, there became a need to develop associated tools to accompany and further advance the use of the existing hardware for intravital imaging. These tools include fluorescent dyes and probes, both generic and specific, and reporter animals to efficiently image cellular structures of interest (**Table 2**).

**Table 2:** Intravital microscopy associated tools.

Generic Fluorescent Dyes			
Subtypes	Target Cell(s)/Process(es)	Disease Application	Sources
TRITC-dextran, FITC-dextran, Texas Red-dextran	Vascular permeability and contrast	Cerebral Microinfarct (Dementia), Stroke, AD	Lee et al., 2021 (59) Neumann et al., 2018 (60) Koffie et al., 2011 (61)
FITC-albumin	Vascular permeability	–	
Rhodamine 6G (62)	Leukocytes, Platelets	Stroke, MS, TBI	Amki et al., 2020 (63) Reichenbach et al., 2015 (64)
Genetic Tags			
Subtypes	Target Cell(s)/Process(es)	Disease Application	Sources
GFP			
*Cx3cr1^GFP/+^*	Macrophage, Microglia	Epilepsy, Stroke, AD	Kim et al., 2011 (65)
*hCD2^GFP^*	T-cells, B-cells	Stroke	Fumagalli et al., 2011 (66)
*DPE^GFP^*	T-cells	CNS Cancer	Mempel et al., 2006 (67)
*Iba1^eGFP/+^*	(Dark) Microglia	Neurodegenerative Diseases, AD	Bisht et al., 2016 (68)
*Pdgfrb^EGFP^*	Pericytes	Neurovascular Diseases. Angiogenesis	Hamilton et al., 2003 (69)
RFP			
*NG2-CreER^TM/td-Tomato^*	Oligodendrocyte Precursor Cells	Stroke	Werner et al., 2023 (70)
*CX3CL1^mCherry^*	Leukocyte Migration and Trafficking	Epilepsy	Kim et al., 2011 (65)
*GAD2-Cre^Ai9Tomato^*	Inhibitory Neurons	Stroke	Latifi et al., 2020 (71)
*Hexb^TdTomato^*	Microglia	CNS Diseases	Masuda et al., 2020 (72)
*Cspg4^DsRed^*	Pericytes	Neurovascular Diseases. Angiogenesis	Zhu et al., 2008 (73)
YFP			
*CD11c^YFP^*	Dendritic Cells	GBM	Ricard and Debarbieux, 2014 (74)
*Thy1^YFP^*	Neurons	Neuron Development and Regeneration, Tumourigenesis, Wound-Healing and Inflammation	Nguyen et al., 2002 (75) Porrero et al., 2010 (76) Jósvay et al., 2014 (77)
Antibodies			
Subtypes	Target Cell(s)/Process(es)	Disease Application	Sources
ICAM-1 (Utilising Nanoparticles)	Endothelial Cells, Leukocytes	Neurovascular Inflammation	Marcos-Contreras et al., 2019 (78)
VCAM-1 (Utilising Nanoparticles)	Endothelium	Stroke, TBI	Marcos-Contreras et al., 2020 (79)
CD45	Lymphocytes	Stroke	Faulhaber et al., 2022 (80)
Ly6G	Neutrophils	MS, Stroke	Kang et al., 2020 (81)
Nanotechnology			
Subtypes	Target Cell(s)/Process(es)	Disease Application	Sources
NanoGd	Phagocytic Cells, Microglia	Stroke	Hubert et al., 2021 (82)
QD	Hematopoietic Cells (T-Cells, Lymphocytes, Monocytes, Macrophages), Amyloid-β	AD	Feng et al., 2013 (83)

Some common generic fluorescent dyes, genetic tags, antibodies and other tools used for preclinical *in vivo* imaging of the brain in various neurodegenerative diseases.

### Conjugated antibodies

The feasibility of chemically conjugating fluorescein-4-isocyanate, a molecule of considerable antigen-binding properties, with antibodies to sensitively label antigens was first described in 1942 (84), and further refined by Coons in 1950 (85). Following suit, Riggs et al. (86) had introduced fluorescein isothiocyanate (FITC) as a more stable fluorochrome. The high susceptibility of FITC to photobleaching (rapid fading subsequent to intense illumination) had since been improved (87) to sustain its popular usage to this day. At a maximum excitation of 498nm, FITC emits a green-coloured fluorescence (518nm); whilst another commonly used fluorochrome, tetramethylrhodamine isothiocyanate (TRITC) (88, 89) emits a red-coloured fluorescence (582nm). The clear colour distinction between this fluorochrome pair has made them a common selection for two-colour fluorescent imaging. The later discovery of isolating phycobiliproteins (PBPs; coloured fluorescent water-soluble proteins) from cyanobacteria and algae in 1982 (90) resulted in the introduction of phycoerythrin (PE; a naturally red-pigment PBP), which is also a popular selection for two-colour fluorescent imaging with FITC. However, FITC, TRITC and PE are large pH-sensitive molecules, and thus are prone to photobleaching (91). This short-coming was overcame with Alexa dyes, which are a series of fluorescent dyes derived from fluorescein, cyanine, aminocoumarine or rhodamine with emission spectra that span the entire visible light range (92). The chemical synthesis of these dyes involves sulfonation (the removal of the hydrogen atom of an organic compound and substituting it with sulfonic acid) that imparts a negative charge to the molecule, making it more hydrophilic and thus improve on water solubility (92). Additionally, compared to the aforementioned parent dyes, Alexa dyes are more resistant to photobleaching and pH changes such that they are able to remain highly fluorescent over pH range of 4–9 (92). Based on these advantages, Alexa dyes which also span the near-ultraviolet (UV), visible light and near-IR ranges, have garnered favourable use in IVM despite being more costly than traditional dyes.

### Fluorescent proteins and reporter animals

Various groups over several decades made progressive development and improvements from the initial discovery of green fluorescent protein (GFP); however, it was the notable works of Osamu Shimomura, Martin Chalfie and Roger Tsien who shared the 2008 Nobel Prize in Chemistry for “the discovery and development of the green fluorescent protein, GFP” that were pivotal in GFP as we know it today. The seminal discovery by Shimomura in 1962 for the identification, extraction and purification of aequorin, a bioluminescent protein from jellyfish *Aequorea Victoria* (93), began it all. The potential of GFP as a tool for *in vivo* protein study remained quiescent until three decades later, when successful cloning and sequencing of the aequorin that flicked the first domino over (94). The solid groundwork made by Prasher (94) allowed Chalfie et al. (95) to be the first to express the coding sequence in prokaryotic *Escherichia coli* (*E.coli*; bacteria) and eukaryotic Caenorhabditis elegans (*C.elegans*; nematode) *in vivo*. Subsequently, the first “green mouse” was developed in 1997 (96). The generation of reporter rodents, particularly mice, that endogenously express fluorescent protein is perhaps the most preferred approach for *in vivo* imaging for their respective advantages of genetic similarity to humans (compared to bacteria and nematode). One example is the development of the *Cx3cr1^GFP/+^* mouse strain, where GFP is expressed on tissue-resident macrophages, including microglia in CNS, and a subpopulation of monocytes in circulating blood (97). Its extensive use in brain IVM for the study of neuroinflammatory processes has contributed to advancements in our understanding of various neurological and cerebrovascular disorders including stroke (98) and Alzheimer’s Disease [AD (99)].

Following the breakthrough of GFP, Matz et al. (100) in 1999 discovered six new fluorescent proteins isolated from non-bioluminescent Anthozoa (class of marine invertebrates) species, expanding the fluorescent protein palette. Of the six, drFP583, a fluorescent protein isolated from the *Discosoma* sp. (anemone) drew considerable attention for its distinct spectral properties to GFP (100). drFP583, now commonly known as DsRed, pioneered commercially available red fluorescent proteins (RFPs). However, the “close-packed” obligate tetrameric structure (101) and slow protein maturation (102) of DsRed became obstacles for *in vivo* imaging. To elaborate further, the red fluorescence of DsRed occurs in a “step-wise” manner shortly after synthesis such that it first proceeds through a green intermediate which exhibits a dim green fluorescence, then a second oxidation reaction is required for the appearance of the red fluorescence. This process has a half-time of approximately 24 hours and thus it requires more than 48 hours for >90% maximal fluorescence (102). In light of this, DsRed was deemed not ideal as an *in vivo* reporter of short-term gene expression or biological processes with narrow time windows. And so began the race to develop an improved monomerised RFP. Establishing the first true RFP monomer, mRFP1, was Campbell et al. (62) of which the development served as the precursor to “mFruits” (a series of second generation mRFP). Despite this, mRFP1 demonstrates a lower photostability than DsRed (62). Of all true RFP monomers, mCherry deserves an honourable mention as it offers the best improvements of both DsRed and mRFP1 such that it has more than 10-fold photostability than mRFP1, faster maturation rate than DsRed whilst also exhibiting the longest wavelength and highest pH resistance (103). Recent works of Latifi et al. (71), provides an example of the use of RFP report mice in brain IVM to enhance our understanding in neurobiology. By crossing Gad2^tm2(cre)Zjh^/J with B6.Cg-Gt(ROSA)26Sor^tm9(CAG-tdTomato)Hze^/J, they successfully label GAD2-expressing inhibitory neurons and led to findings that suggest neuronal network topology alters following stroke.

Notably, cellular processes and movements *in vivo* are often complicated, and stable expression of endogenous fluorescent proteins may not provide significant insights into the dynamic activities. For this reason, the development of transgenic mice expressing photoconvertible fluorescent protein such as Kaede may help to fill the void (104). Kaede, cloned from the stony coral *Trachyphyllia geoffroyi*, emits bright green fluorescence after synthesis, but changes efficiently to a bright and stable red fluorescence on irradiation with UV or violet light (105). Whilst not currently readily used in brain IVM yet, future use of photoconvertible proteins to track movement of cells in the CNS will significantly contribute to our understanding of host responses in the context of various neurobiological diseases.

### Quantum dots – a small but powerful future for IVM

Excitingly, the 2023 Nobel Prize in Chemistry was awarded to Moungi G. Bawendi, Louis E. Brus and Alexei I. Ekimov for the seminal development of quantum dots (QD) – tiny semiconductor nanocrystals or nanoparticles that possess both photoluminescent and electroluminescent properties (106–108). Whilst this technique is not broadly implemented across the brain IVM field, we believe QDs may provide a platform for achieving greater imaging depths and resolution in visualising the biological activities in the deep brain striatum.

The nanoparticles contain a core, which is made of a heavy metal cadmium compound. Issues with QD optical properties and cytotoxicity were overcome by surface modification of the cores with organic (108) and inorganic capping (109–114). More importantly, the biological application of QD gained traction through the work of Bawendi and colleagues (108) who identified that the hydrophobic ligands present on the nanocrystal surface, trioctylphosphine (TOP) and trioctylphosphine oxide (TOPO), can be substituted with water-soluble hydrophilic bioconjugates whilst retaining QD surface integrity. The first biological applications of QDs were reported in *in vitro* cell culture (115, 116) and later in *in vivo* imaging of *Xenopus* embryos (117) and mice (118–120). QDs have since evolved to be an attractive fluorescent probe in research for its high quantum yield, particularly achieved by multiple zinc sulphide (ZnS) layers (113, 114), photostability (121, 122), broad absorption (excitation), narrow and symmetrical emission wavelength (123, 124). Since its development, QD-based research has particularly focussed around cancer imaging (125) and diagnostics (126), but is yet to be stable in brain IVM research. Despite this, work combining 3PM with QDs has facilitated imaging vasculature and subsequent blood flow in the brain at 2 mm deep (127). In particular, conjugation of amyloid-beta (Aβ) has allowed for successful *in vivo* molecular imaging of AD in mice (83), demonstrating the promising use of QD in the field of neurobiology for not only *in vivo* imaging and diagnostics, but also theranostics.

Specifically for neurobiology, reporter mice have been a gold-standard in assessing tissue-resident cells, bypassing restrictions of QD or antibodies crossing an intact BBB. GFP and RFP fluorochromes maintain dominance in the IVM field, and this is no different in brain IVM. Excitation of fluorochromes in the UV or violet spectrum are associated with unwanted tissue damage by UV radiation. Conversely, fluorochromes in the far-/infra-red spectrum are readily excited but insufficiently bright enough for deep tissue imaging, despite advancements in far-/near infra-red genetically altered animals (128). The limitation of available colours for reporter mice limits research toward cell-cell interaction in the brain. Advancements in generating more cell-specific, optically preferential genetically encoded reporter mice is where the future of brain IVM lies.

## IVM: application in neurobiology

Brain IVM and its associated tools have been an invaluable technology at the forefront of imaging the real-time dynamic cellular behaviour and interactions *in vivo* during cerebral disease. In this section, we will highlight the use of brain IVM in studying some of the cell-cell interactions involved in the pathological processes of neuroinflammation.

### Microglia

Microglia are one of the most studied cells in neuropathology as they are generally the first brain-resident responders following brain injury. During infection-driven neuroinflammation, microglia are shown to have functions in pathogen phagocytosis, astrocyte activation and release molecules that recruit cells from the periphery into the brain (129, 130). Brain IVM has been instrumental in revealing microglia inflammatory activity by assessing their morphological changes and migratory activity. The cell shape of a naïve, surveillant microglia is inherently ramified with numerous dendritic filopodia, however this changes dramatically following their activation by inflammatory stimuli. The cell body of activated microglia is enlarged, and coupled with reduced dendrites, branches, and branch length (131–133), thus the cell shape of these cells resembles an ameboid structure. These dynamic cell shape changes of microglia are often missed in brain slices with single timepoint ‘snapshot’ *ex vivo* analysis. As such, real-time *in situ* analysis of microglia behaviour provides a greater understanding of the immune activity during neuroinflammatory processes. In fact, a recent perspective by Paolicelli et al. has defined numerous sub-populations of microglia, including disease-associated, interferon-responsive, glioma-associated, lipid droplet-accumulating and proliferation-region-associated (133), with many of the recent discoveries made through the innovative use of brain IVM coupled with ‘omics’ analysis.

Though most knowledge pertaining to microglia response is derived from both confocal and 2PM, the latter has more recently become the gold-standard in monitoring microglial dynamics following neuroinflammation *in vivo*. Brain intravital confocal microscopy was traditionally facilitated through the implantation of a cranial window to overcome light penetration impairment by the skull. However, recent revelation into the pathological role of cells within the dura, cerebral spinal fluid cavity and calvarium bone marrow suggest this may manipulate the neuroinflammatory response (13, 134). Therefore, the ability to image past a thinned, but intact, skull with 2PM is more preferential for long-term imaging of deeper cellular structures with reduced phototoxicity and increased depth penetration. To identify microglia by brain IVM, reporters used include *Cx3cr1^GFP/+^* (65, 131, 135, 136), *Iba1^eGFP/+^* (137) and *Hexb^TdTomato^* (72) (**Table 2**). Other fluorescent reporter mouse strains are used but are generally for *ex vivo* analyses due to low fluorochrome strength.

In addition to considering which transgenic reporter mouse strain to use for brain IVM, it is also important to understand the potential limitation of imaging capabilities with some of the preclinical disease models. The nature of neuroinflammatory disease is dynamic, therefore events may occur deep in the striatum ventral region, presenting a difficult area for 2PM to access *in vivo*. In contrast, brain peripheral regions such as the sensorimotor cortex is accessible within the capability of 2PM *in vivo*, and is generally favoured for brain IVM (60, 138). Besides the use of transgenic animals for studying microglia dynamics and behaviour *in vivo*, NanoGd is a novel multimodal nanoprobe that is specifically designed to be internalised by phagocytic cells. Very recently, the combination of transgenic reporter animal model *Cx3cr1^GFP/+^* mouse strain with NanoGd was developed to advance preclinical longitudinal *in vivo* imaging of microglia/macrophage phagocytic function under ischemic conditions (82). In this study, brain IVM with 2PM revealed sham‐operated *Cx3cr1^GFP/+^* mice injected with NanoGd showed no morphological evidence of CX3CR1‐GFP^+^ cell activation, negligible NanoGd leakage in the brain parenchyma and very little NanoGd internalisation by parenchymal CX3CR1‐GFP^+^ cells. Following brain ischemia, NanoGd was observed in the parenchyma surrounding the infarct core and internalisation of the extravasated NanoGd by CX3CR1‐GFP^+^ cells was evident, strongly suggestive of reduced vascular integrity and enhanced microglia/macrophage phagocytic activity. This method appears to be a promising tool for studying the spatiotemporal response of microglia/macrophages at the ischemic lesion, with the advantage to gain an insight into their level of phagocytic function.

### Neutrophils

Widely accepted as the first brain-infiltrating peripheral immune cell in response to neuroinflammation, neutrophils play a pivotal role in acute inflammation prognosis (139). Brain IVM unveiled a loss of BBB integrity, coupled with leukocyte extravasation through a chemotactic-driven migration facilitates the filtration of neutrophils into brain parenchyma during acute neuroinflammation (60, 140, 141). Traditionally, imaging intravascular neutrophils by confocal or 2PM intravital microscopy is simply conducted following the intravenous administration of fluorophore-conjugated antibodies against Ly6G (1A8 clone), which does not impact neutrophil recruitment during acute inflammation (142). However, neutrophils are shown to adhere in capillary segments distal to the ischemic clot to stall blood flow and cause “no-reflow” in cerebral penumbra (63, 143). This has major implication in relying on intravascularly delivered fluorophore-conjugated antibodies to study the identity of cells infiltrating into the post-stroke brain using IVM. This is furthered as prolonged exposure to anti-Ly6G antibodies leads to long-term neutrophil depletion (144, 145). These limitations were overcome using genetically modified reporter mice. In the past, *LysM^eGFP/+^* mice (146) that endogenously express enhanced green fluorescent granulocytes has been used in various *in vivo* neutrophil research (147), however this reporter mouse is not neutrophil-restricted. The expression of LysM is common in myeloid cells, thus GFP-positive monocytes and other myeloid cells were observed.

In light of this, the *Catchup^IVM^* mouse was developed to enable strong neutrophil-specificity, without labelling other myeloid cells. This was achieved by genetic alternation of the Ly6G locus to create Cre-T2A-tdTomato construct and further amplify the endogenous red fluorescent signal by breeding with ROSA (148). Moreover, Neumann et al. developed a novel mouse model that involved crossing the *Catchup^IVM^ mice* with *Cx3Cr1^GFP/+^* mice in which 2PM brain IVM of this new mouse strain would allow for the visualisation of tdTomato-positive neutrophils interacting and engulfed by GFP-positive microglia/macrophages in the injured ischemic brain parenchyma (60). Whilst it is known that microglia phagocytose brain-infiltrating neutrophils (60), it is anticipated that future studies with novel mouse strains and innovative imaging technologies will support a better understanding of the neutrophil-microglial interaction during neuroinflammation and reveal new targets to modulate the neuroinflammatory response.

### Lymphocytes

Unlike neutrophils, which are dominant during settings of acute inflammation, brain-infiltrating lymphocytes are more commonly observed during chronic, autoimmune-driven neurological diseases. In an ever-growing field of identifying novel lymphocyte sub-populations, the technique for their observation by IVM is modest. To our knowledge, the first assessment of T lymphocyte activity in the brain was conducted in 2002 by Piccio et al., whereby peripheral lymphocytes were stained with simple molecular probes after isolation, adoptively transferred to recipient mice for subsequent IVM on the brainstem (149). This process was later developed to capitalise on lymphocyte antigen presentation (e.g. CD4/CD8) to isolate, sort and manipulate lymphocytes from fluorescent mice (e.g. *β-actin* ^GFP^) and fluorescent antigen-specific T-cell strains (e.g. *OT-1* or *P14*) by IVM. Hereafter, the activity of CD4+T Cells (150) and the interaction between lymphocytes and CNS-resident myeloid cells [*Cx3cr1^GFP/+^* (151)] have been characterised during models of multiple sclerosis (experimental autoimmune encephalitis) via IVM. This technique has been mirrored during models of CNS cancer, parasite infection and viral infection (152–154), and further innovated by assessing peptide-MHC-complexes in adoptively transferred CD8+ T cells through the generation of genetically coded calcium indicator, GCaMP6s (153).

A result of the ever-expanding lymphocyte library creates difficulty in generating a specific, targeted fluorescent lymphocyte reporter mouse for IVM assessment. This was first attempted using the human CD2 promotor, to generate the *hCD2* ^GFP^ mouse, which is GFP-positive in (most) T cells and some B cells (155). This mouse has been used to track lymphocyte infiltration into the brain post-stroke by 2PM IVM (66), however a lack of specificity to T cell sub-populations makes it difficult to draw conclusions regarding T cell polarisation. A second mouse was made for greater specificity utilising the CD4 promoter, the *DPE* ^GFP^ mouse (67). This mouse has greater specificity to T cells, but also perivascular macrophages [albeit their drastic difference in morphology makes for easy distinction (156)]. Using 2PM, this mouse was utilised to identify T cell adhesion in the cerebral endothelium during malaria initiation (157). Despite the availability of these reporter mice, they are infrequently used due to their lack of T-cell specificity, especially in the context of brain IVM. Until a global polyclonal T-cell-restricted fluorescent reporter mouse is developed, the current ‘gold-standard’ remains in adoptive transfer of antigen-specific T-cells.

### Neurons

Neurons are nerve cells critical for normal bodily function. In the setting of neuroinflammation, neurons are highly susceptible to severe cellular damage and death due to exposure to hypoxia and damage associated molecular pattern-driven inflammation. Neurons have diverse functional properties; therefore, their classification is important to understand their involvement in disease progression. Similar to microglia, neurons are defined by morphological, physiological and molecular criteria (158, 159). Studying these cells during neuropathology is a challenge due to the complex brain microenvironment which create issues in isolating intact neurons for profiling. However, this is combatted utilising brain IVM.

Traditionally, the gold-standard of neuron reporter mice was the *thy1^XFP^* (collective term used for cyan-, yellow-, red- or green-fluorescent protein) reporter (160), which demonstrates fluorescence over centimetres of axons and millimetres of dendrites. This was furthered by the development of the *Brainbow* mouse (161), which has combinatorial XFP expression to identify individual adjacent neurons and the origin of each dendrite. The Allen Institute for Brain Science has since reported 53 novel reporter mice for specific neuron identification and characterisation (162, 163). Using the *thy1^XFP^* mouse, Sigler et al. demonstrated acute neuronal loss post-sterile neuroinflammation, but revealed elevated rates of synaptogenesis within recovering peri-infarct tissues (164).

More recently, developments in techniques have facilitated assessment of neuronal activity through calcium imaging by 2PM. This is based on the knowledge that active, ‘firing’ neurons has elevated levels of intracellular calcium, which can be detected by fluorescent probes (165). For example, the functional connectivity of the neuronal network during stroke recovery has been demonstrated via *in vivo* calcium imaging and 2PM (166), using the Thy1-GCaMP6s mouse, which genetically express a GFP calcium indicator in excitatory neurons (167). Coupled with neuron reporter mice [GAD2Cre/Ai9Tomato, described in Latifi et al. (71)] these studies identified that photothrombotic stroke causes disruptions within the inhibitory circuits, which were not compensated for by surviving neurons, indicating that the brain does not remap neuronal networks post-ischemic stroke (166).

### Pericytes

Cerebral vasculature is notability distinct from vasculature of other organs, in that there is a neurovascular unit. Pericytes are found throughout the body, however they are often coupled with vascular smooth muscle cells, and credited for the development and maintenance of BBB integrity (168) and neurovascular angiogenesis (169) - the generation of vasculature from pre-existing vasculature. Sprouting and immature vessels secrete an abundance of platelet-derived growth factor (PDGF)-B to recruit BBB-supporting cells, including pericytes, and facilitate their growth. With this in mind, PDGF receptor β (PDGFRβ) is routinely used in immunohistochemistry to identify pericytes. However, an intact BBB disables intravenously administered antibody identification of tissue-resident cells; subsequently the *Pdgfrb* ^EGFP^ transgenic mouse was generated (69). NG2 proteoglycan (also known as chondroitin sulfate proteoglycan-4 (CSPG-4)), a co-receptor of PDGF, is also a popular marker for identifying pericytes (170), hence the use of a second reporter mouse *Cspg4* ^DsRed^ (73). Importantly, markers such as PDGFRβ and NG2 are not limited solely to pericytes and are shared by sub-populations of perivascular fibroblasts and macrophages. To overcome this, specific anatomical location is key in defining pericytes, and this was further combated by the dual-reporter *Pdgfrb* ^EGFP^ *Cspg4* ^DsRed^ mouse (171).

This being said, intravital imaging of brain-resident pericytes is currently extremely limited. The relatively low abundance of PDGFRβ and NG2/CSPG-4 on pericytes leads to poor fluorescence in reporter mice, and therefore has restricted its use in intravital imaging. Whilst this perhaps is not a major issue for most organs, the roadblock added by the skull creates severe difficulties in imaging brain-resident pericytes. This was recently impressively navigated in a longitudinal study of the *Cspg4* ^DsRed^ mouse following cerebral infarction which required the use of a glass intracranial window (59). Nevertheless, significant advancements are necessary to improve reporter specificity and fluorochrome strength toward improved pericyte research.

### Vasculature and its integrity

To image and study cerebral vasculature, brain endothelium is often labelled with fluorophore-conjugated antibodies. Due to the ease of access of the antibody to the antigen on endothelial cells by intravenous injection, vasculature-reporter mice are rarely used for brain IVM. Indeed, intravenously delivered dyes are more often used to assess vascular integrity in the development of neuroinflammatory disease. A loss of vascular integrity is not only indicative of peripheral inflammatory cell infiltration into the brain, but also the extravasation of DAMPS such as fibrin(ogen) (172, 173). Cerebral microvascular integrity is regularly studied *in vivo* with 2PM following intravenous injection of fluorescence-conjugated dextrans. There is a range of commercially available fluorescence-conjugated dextrans in different sizes, available up to 2,000 kDa, whereby extravasation implies degrees of reduced BBB integrity. Evans blue dye has been widely used for its absorption at ~620 nm as a stable vascular contrast agent. The high water solubility, slow excretion rate and high-affinity binding nature to plasma albumin are properties that bequeathed its extensive use in defining vascular perfusion (174). Importantly due to their small size, dextrans and vascular dyes are cleared relatively quickly by most organ, therefore they are not suitable for longitudinal studies.

The knowledge of the neuro-pathophysiology of neuroinflammation including, but not limited to, vasculature structure and metabolism has suffered from limitations of available intravital imaging technologies. PAI harnesses its high sensitivity to hemoglobin, which is a widely abundant biological protein in the bloodstream, to attain wide applications in angiography (175). In the context of preclinical research, PAI has recently been advanced to perform structural and functional imaging of ischemia-affected deep brain regions. As demonstrated, PAI has increasing potential in research and diagnosis, particularly for its impressive image acquisition of vasculature (18, 176, 177). With the efforts of advancing the raster scanning of PAM into higher scanning speed alternatives (52–57) the development of ultrafast functional photoacoustic microscopy [UFF-PAM (18)] demonstrated the capability of imaging changes in the microvasculature of the brain during and post-neuroinflammation. By monitoring the wave of morphological changes in cells within the grey matter of the CNS, known as a spreading depolarisation (SD) wave, and its impact on cerebral hemodynamics, UFF-PAM was able to confirm that the SD waves had the capacity to vasoconstrict and exacerbate ischemia (18).

## Conclusions

Whilst the humble beginnings of IVM began nearly two centuries ago, technological advancements in recent decades have driven an exponential growth and improvement in the hardware, tools and applications of brain IVM. Advancements in brain IVM have greatly enhanced our knowledge of neurobiology, and disease progression following various neuropathology. In this review, we have provided an overview of recent studies that effectively utilised brain IVM to better our understanding of brain pathophysiology. Imaging and studying cell-cell interactions with novel transgenic mouse stroke led to the discovery of new molecular pathways that mediate changes in resident cell activity and immune cell recruitment which promote an inflammatory microenvironment. The imaging depth limitation of 2PM reduces our ability to accurately examine the inflammatory core following the clinically-relevant models of neuroinflammation model. However, this may perhaps be overcome by 3PM in due course. Excitingly, the potential opportunity to image through the adult mouse skull in awake mice with 3PM will provide unprecedented insights into the neural pathways that mediate sensory and motor recovery in active post-stroke animal. The future for brain intravital microscopy is certainly ‘bright’.

## Author contributions

AS: Data curation, Formal analysis, Investigation, Writing – original draft, Writing – review & editing. CW: Conceptualization, Funding acquisition, Resources, Supervision, Writing – review & editing. JB: Conceptualization, Formal analysis, Funding acquisition, Supervision, Writing – original draft, Writing – review & editing.
